# Supplementary material for: Methods of performance analysis in women’s Australian football: a scoping review
Source: PeerJ. 2023 Mar 13;11:e14946. doi: 10.7717/peerj.14946 (PMC10019326; doi:10.7717/peerj.14946)
Supplement: Supplemental Information 2 [file peerj-11-14946-s002.docx]

|  | Search Terms |
| --- | --- |
| Population | *“Women*” or “Female” or “Girl*”* |
|  | ***AND*** |
| Concept | *“Australian Football” or “AFLW” or “Australian Rules Football” or “AFL Women”* |
|  | ***AND*** |
| Context | *“performance analy*” OR “notational analy*” OR “match analy*” OR “game analy*” OR “patterns of play” OR “game styles” OR “performance indicators” OR “dynamic systems” OR “systems analy*” OR “sports analy*” OR “team behaviour” OR “physical demands” OR “match demands” OR “running” OR “conditioning” OR “fitness” OR “physiology” OR “techn*” OR “tacti*” OR “skill*”* |

Supplement 2: Search Terms Used
